# Repositioning Natural Products in Drug Discovery

**DOI:** 10.3390/molecules25051154

**Published:** 2020-03-04

**Authors:** Giulio Rastelli, Federica Pellati, Luca Pinzi, Maria Cristina Gamberini

**Affiliations:** Department of Life Sciences, University of Modena and Reggio Emilia, Via Giuseppe Campi 103, 41125 Modena, Italy; federica.pellati@unimore.it (F.P.); luca.pinzi@unimore.it (L.P.); mariacristina.gamberini@unimore.it (M.C.G.)

Drug repositioning (o repurposing) has become one of the most popular and successful strategies to reduce failures typically associated with drug discovery [1]. It involves the identification of novel biological targets and different therapeutic uses of already approved and/or investigational drugs, including drugs that did not meet the primary therapeutic expectations. As such, many preclinical development and optimization issues, including adverse toxicology profiles, can be prevented or at least minimized. Although most drug repurposing success stories derived from serendipity, current research efforts focus on predicting repurposing possibilities on rational grounds [2]. Interestingly, while most drug repurposing campaigns rely on compounds derived from chemical synthesis, natural products can provide significant opportunities. Natural products are characterized by unique and favorable properties, a significant structural diversity, and by a high number of pharmacological activities [3]. Therefore, they are privileged chemical entities for drug (re)discovery strategies, which can highlight novel therapeutic utilities potentially unrelated to their original biological space [4]. Natural products have recently seen a resurgence of interest in drug discovery, but the way this is happening is significantly different from the past. Newer and emergent technologies, such as computational screening, proteomics, metabolomics and big data analysis have come into play to drive and speed up the “repurposing” of natural compounds and, more generally speaking, of nature-inspired compounds.

In light of all the above, this Special Issue will focus on *computational* and *experimental* approaches in the research area of repurposing natural products, including studies on, but not limited to, the identification of new biological targets of natural compounds, the discovery of bioactive natural and/or nature-inspired compounds by in silico screening, their isolation and characterization, de-replication of natural extracts, analysis of structure–activity relationships of natural bioactive compounds. 

Overall, this collection will provide a multi-disciplinary view of how different research fields can interact in either the discovery or the repurposing of bioactive nature-inspired compounds. This issue is connected to the “*Repositioning Natural Products in Drug Discovery*” meeting held at the University of Modena (Italy) on Jan 17th, 2020 (http://www.mmddlab.unimore.it/site/home/rnpdd-meeting.html).
